# RETRACTION: LncRNA SLNCR1 Facilitates Angiogenesis and Tumor Growth in Melanoma via DNMT1‐Mediated Epigenetically Silencing SPRY2

**DOI:** 10.1111/srt.70184

**Published:** 2025-05-16

**RETRACTION**: K. Li, L. Wu, and J. Jiang, “LncRNA SLNCR1 Facilitates Angiogenesis and Tumor Growth in Melanoma via DNMT1‐Mediated Epigenetically Silencing SPRY2,” *Skin Research and Technology* 30, no. 9 (2024): e13910, https://doi.org/10.1111/srt.13910.

The above article, published online on 19 September 2024 in Wiley Online Library (wileyonlinelibrary.com), has been retracted by agreement between *Skin Research and Technology*; and John Wiley & Sons Ltd. Following publication of the article, several flaws and inconsistencies between results presented and methodology described were found. Furthermore, the article lacks important information to interpret and reproduce the findings. The material provided by the authors upon request did not adequately address the concerns. Therefore, the decision to retract this article was taken as the editors consider its conclusions invalid. The authors have been informed of the decision of retraction and disagree with it.

